# Surgical Low-Value Care Between Fee-For-Service and Salaried Health Care Systems

**DOI:** 10.1001/jamanetworkopen.2025.46213

**Published:** 2025-12-02

**Authors:** Andrew J. Schoenfeld, Kaitlyn E. Holly, Madison N. Cirillo, Aaron W. Gu, Malina O. Hatton, Joshua M. Coan, Christian L. Coles, Tracey P. Koehlmoos

**Affiliations:** 1 Department of Orthopaedic Surgery, Mass General Brigham, Harvard Medical School, Boston, Massachusetts; 2 Center for Health Services Research, Uniformed Services University of the Health Sciences, Bethesda, Maryland; 3 United States Naval Academy, Naval Academy, Maryland; 4 Harvard Medical School, Boston, Massachusetts

## Abstract

**Importance:**

Low-value care has been recognized as a pernicious phenomenon that increases health care costs and contributes to suboptimal care delivery. Low-value surgery may be less likely in systems that used salaried reimbursement as opposed to fee-for-service.

**Objective:**

To explore the association of reimbursement model with low-value surgery among a battery of elective procedures.

**Design, Setting, and Participants:**

This cohort study used TRICARE health care claims to compare rates of low-value surgery over fiscal years 2016 to 2023. Participants included patients aged 10 years and older who underwent acromioplasty, partial knee meniscectomy, shoulder rotator cuff repair, wrist arthroscopy, or ankle arthroscopy. Data were analyzed from January to May 2025.

**Exposure:**

Direct vs private sector care.

**Main Outcomes and Measures:**

The primary outcome was the comparison of low-value care in patients in direct care vs private-sector care. An interaction between environment of care and year of surgery was retained in all models. Multivariable logistic regression analyses were used to adjust for case mix. Secondary analyses were limited to non–active-duty individuals to account for differences in low-value care for each surgical procedure.

**Results:**

A total of 304 908 procedures were included. The mean (SD) patient age was 47.2 (12.9) years, with 189 648 (62%) male patients. Partial meniscectomy was the most common surgical procedure (128 363 procedures [42%]), followed by acromioplasty (87 721 procedures [29%]). The percentage of low-value surgery in direct care was 20%, compared with 35% in the private-sector (χ^2^_2,304 908_ = 90007.01; *P* < .001). After adjusting for case mix, the private sector demonstrated significantly greater odds of low-value surgery (odds ratio [OR], 1.41; 95% CI, 1.38-1.45). Low-value surgery was significantly lower in each respective sector for 2020 to 2023 compared with 2016 to 2019 (direct care: OR, 0.78; 95% CI, 0.73-0.83; private sector: OR, 0.93; 95% CI, 0.91-0.96).

**Conclusions and Relevance:**

In this cohort study of 304 908 surgical procedures, direct care evinced a significantly lower likelihood of low-value surgery in both 2016 to 2019 and 2020 to 2023. These findings support the contention that changing clinician reimbursement models from fee-for-service to salaried is associated with lower rates of low-value care.

## Key Points

**Question:**

Is a health care system’s reimbursement model associated with provision of low-value surgical care?

**Findings:**

This cohort study including 304 908 procedures identified significant differences in the use of low-value care in a salaried direct care environment (20%) compared with the private-sector fee-for-service setting (35%). Low-value surgery was significantly lower in each respective sector for 2020 to 2023 vs 2016 to 2019.

**Meaning:**

This cohort study supports the contention that changing clinician reimbursement models from fee-for-service to salaried was associated with lower rates of low-value care.

## Teaser

This cohort study explores the rates of low-value surgical procedures by reimbursement model and over time.

## Introduction

Over the last 15 years, low-value care has been recognized as a pernicious phenomenon that increases health care costs and contributes to suboptimal care delivery in medicine as a whole, as well as various surgical subspecialties.^1,2,3,4,5,6^ In 2019, the estimated annual waste from low-value care was reported to be in the range of $76 to $100 billion per year.^5^ Within the field of surgery, low-value care is thought to largely derive from variations in physician beliefs about surgical indications, lack of evidence supporting the ideal treatment approach, patient preferences for surgical treatment and their associated beliefs regarding the impact of surgery on outcomes, as well as clinician-induced demand.^3,4,6^ In the latter phenomenon, for various reasons both implicit and explicit, surgeons may influence patients to undergo surgical procedures that do not have proven efficacy or that may not be indicated in the patients’ particular health care context.^3,4,6,7^ Such low-value interventions increase costs and the risk of complications, as well as other associated adverse events, without a high likelihood of benefit at baseline.^2,5^

Recent studies have emphasized that low-value surgical care is less likely to be present in larger, tertiary facilities and in systems that use a salaried reimbursement model as opposed to a fee-for-service model.^1,4,6^ Within the US, the Military Health System (MHS) has been an attractive setting for the study of low-value care, given it possesses both salaried (ie, direct care: treatment is delivered by facilities operated by the Department of Defense) and fee-for-service (ie, private-sector care: treatment is delivered through civilian facilities using the TRICARE insurance product) environments in which health care is delivered to a population that has been shown to be representative of the US population aged 18 to 64 years on racial, socioeconomic, educational, and vocational grounds.^4,6,8,9,10^ While some research conducted within the MHS has highlighted the potential for low-value care to be mitigated in a salaried health care system, findings have been restricted to relatively rare procedures with limited market penetration on the direct care side (eg, carotid endarterectomy and lumbar spine surgery^4,6^), resulting in impaired generalizability.

In this context, we sought to explore the influence of reimbursement model on low-value surgical care using a broader range of elective interventions that are more commonly performed among MHS beneficiaries in both the private sector and direct care. These include arthroscopic procedures of the knee and shoulder, which alone make up close to 1.5 million surgical interventions in the US each year.^11^ These surgeries are also among the most commonly performed procedures in the population covered by the MHS.^12,13,14^ As a result, we believe that the findings from the current line of research have greater translational capacity to the MHS and the broader US health system as a whole. We hypothesized that the prevalence of surgical low-value care would be lower in the salaried (direct care) environment than it would in the fee-for-service system (private sector).

## Methods

This cohort study was deemed exempt by the institutional review board of the Uniformed Services University with waiver of consent because it used deidentified claims-based data that were collected in the course of regular health care delivery. This study is reported in line with the Strengthening the Reporting of Observational Studies in Epidemiology (STROBE) reporting guideline.

### Data Source

We used TRICARE health care claims from the MHS Data Repository for fiscal years 2016 to 2023. MHS data have been successfully used in the past to examine aspects of surgical care delivery, postoperative outcomes, and health care policy.^2,4,6,10,12,13^ The means through which MHS data are collected, made available, accessed, and analyzed have been described elsewhere.^8,12,13,14^ Importantly, MHS data do not capture procedures performed through the Veterans Health Administration or combat-related surgical procedures.

For the purposes of this investigation, we focused on a battery of surgical procedures, including shoulder acromioplasty, partial knee meniscectomy, shoulder rotator cuff repair, wrist arthroscopy, and ankle arthroscopy. These procedures were selected as they have a well-defined literature identifying specific aspects of low-value care,^1,15,16,17,18,19,20,21,22,23,24,25^ are widely used in both the direct care and private sector environments of the MHS,^12,13,26^ and have translational capacity to other types of elective surgery.^2,12,13,14^

We identified patients aged 10 years and older who underwent outpatient surgery using Current Procedural Terminology codes. Using *International Statistical Classification of Diseases and Related Health Problems, Tenth Revision* (*ICD-10*) diagnostic codes, we excluded all procedures with a general diagnosis of infection, tumor, or trauma at the time of the surgery. We then reviewed each surgical procedure and excluded procedures in association with infection, tumor, or trauma involving the shoulder, knee, wrist, or ankle. (eAppendix in Supplement 1).

For each surgical procedure, we documented whether the surgery was performed at a facility maintained by the Department of Defense (direct care) or a civilian health care organization (private sector) and the fiscal year of the surgery (2016-2019 or 2020-2023). Sociodemographic and clinical data identified for patients included age at the time of surgery, biologic sex (female, male), race (based on self-report; recorded as Black, White, other [including American Indian or Alaskan Native, Asian or Pacific Islander, and other race], or missing/unknown), medical comorbidities (identified using the Charlson Comorbidity Index^27^ and categorized as none, 1, 2, or ≥3), beneficiary status (active duty, retired, dependent, other, unknown), hospital region (Northeast, Midwest, South, West, other [including US territories and international]), and sponsor rank (enlisted junior, enlisted senior, junior officer, senior officer, other, unknown). In studies using MHS claims, sponsor rank has been shown to be a useful proxy for socioeconomic status, with junior enlisted indicative of a lower socioeconomic background.^8,10,12^ Time period was dichotomized as 2016 to 2019 and 2020 to 2023 to account for the impact of the COVID-19 pandemic and changes in health care delivery within the MHS, including introduction of a new medical record and transition to the Defense Health Agency.^14^ All variables, with the exception of race, exhibited rates of missingness less than 1%. Dependent race is frequently underreported in the MHS due to it not being a mandatory requirement, and 23% of our study population had a missing race variable. In line with prior recommendations for handling missing or unknown race in MHS data, we used the reweighted estimating equations method to account for missing or unknown patient race.^8,28^

### Definition of Low-Value Care

We defined low-value surgical care using the following literature-based criteria^1,15,16,17,18,19,20,21,22,23,24,25^ for each of the included procedures: acromioplasty alone for patients older than 18 years, partial meniscectomy alone for patients older than 40 years, arthroscopic wrist debridement alone for patients older than 40 years, ankle arthroscopy alone for patients older than 40 years, and rotator cuff repair alone (with or without arthroscopic assistance) for patients older than 40 years. These literature-based definitions are largely predicated on the performance of these procedures in specific age groups, randomized trials, and the higher risk of failure or need for further additional interventions beyond the index procedure in certain populations. When other procedures were performed in addition to potentially low-value interventions within the same joint at the time of the index event, these were not considered low-value care. As such, even when certain procedure codes are typically only supported in combination with other interventions, these were still included in our search strategy for holistic assessment.

### Statistical Analysis

Our primary outcome was low-value surgical care, with the care setting (ie, direct care vs private-sector care) the primary explanatory variable. All other variables extracted were considered covariates for the purposes of adjusting for case mix. Initially, we divided procedures into 3 categories: procedures performed alone on patients within the age criterion (low-value care), procedures performed alone on patients outside the age criterion, and procedures performed in conjunction with other surgical interventions. In regression analyses, the latter 2 categories were collapsed into a single group that was defined as non–low-value care.

Cross-tabulations were used to examine demographic and clinical characteristics using χ^2^ tests. Multivariable logistic regressions were conducted to estimate the probability of low-value care and adjusted for case mix. In regression testing, the direct care setting was used as the reference group for the environment of care, and an interaction test between environment of care and year of surgery (2016-2019 vs 2020-2023) was included in all regression analyses. As indications and motivations for surgical intervention may be different in the active-duty population, we performed secondary analyses limited to non–active-duty individuals within each of the specific surgical procedures. The results of regression tests were reported using odds ratios (ORs), 95% CIs, and *P* values. Bonferroni correction was used for interaction terms to minimize familywise error rates. Statistically significant results were considered those with 2-sided *P* < .05 and 95% CIs exclusive of 1 following multivariable logistic regression. All analyses were performed using SAS software version 9.4 (SAS Institute). Data were analyzed from January to May 2025.

## Results

We identified 304 908 procedures over the time period studied. Overall, patients had a mean (SD) age of 47.2 (12.9) years, with 189 648 (62%) male; 37 127 patients (12%) were Black, 172 391 patients (57%) were White, and 25 011 patients (8%) identified as other race (eTable in Supplement 1). Overall, partial meniscectomy was the most common surgical procedure (128 363 procedures [42%]), followed by acromioplasty (87 721 procedures [29%]). Across each individual year of the investigation, the volume of surgical interventions showed a general reduction over time (Figure, A) that was mirrored in the provision of low-value care (Figure, B). We found that 98 150 procedures (32%) met criteria for low-value care; among these, 54 553 low-value procedures (56%) were performed in 2016 to 2019 compared with 43 597 low-value procedures (44%) in 2020 to 2023 (*P* < .001) (Table 1). Of the final cohort, 233 133 procedures had complete data and were used in regression analyses. After adjusting for case mix, multivariable analysis found that procedures performed in the private sector had significantly greater odds of meeting criteria for low-value care (OR, 1.41; 95% CI, 1.38-1.45; *P* < .001) (Table 2).

**Figure.**
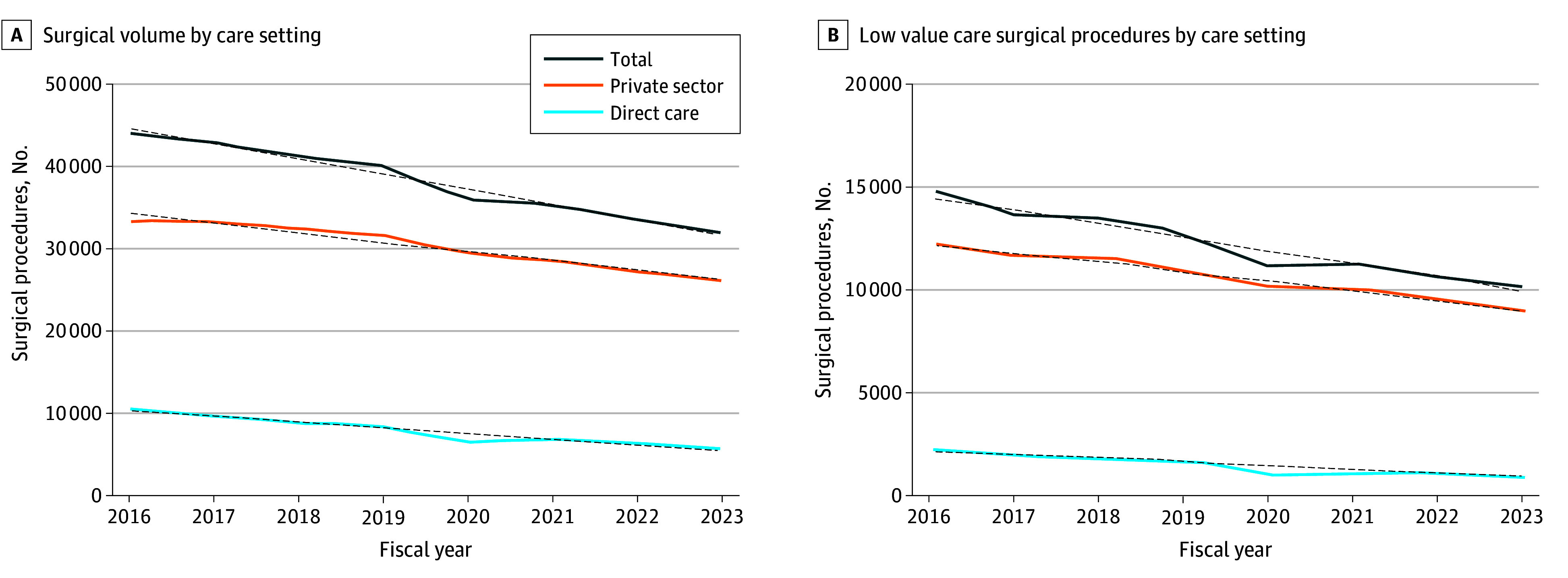
Total Surgical Volume and Low-Value Surgery by Care Setting Dotted lines indicate linear trends.

**Table 1.** Sociodemographic and Clinical Characteristics of the Study Population Stratified by Care Setting

Characteristic	Low value procedures, No. (%)			χ^2^ *P* value
	In direct care	In private sector	Totals in the military health system	
Total	12 699 (13)	85 451 (87)	98 150 (100)	NA
Time period				
2016-2019	8030 (63)	46 523 (54)	54 553 (56)	<.001
2020-2023	4669 (37)	38 928 (46)	43 597 (44)	
Type of surgery				
Acromioplasty	2199 (17)	17 286 (20)	19 485 (20)	<.001
Ankle arthroscopy	504 (4)	1038 (1)	1542 (2)	
Partial meniscectomy	7783 (61)	49 145 (58)	56 928 (58)	
Rotator cuff repair	2002 (16)	17 091 (20)	19 093 (19)	
Wrist arthroscopy	211 (2)	891 (1)	1102 (1)	
Patient sex				
Female	3570 (28)	39 667 (46)	43 237 (44)	<.001
Male	9129 (72)	45 784 (54)	54 913 (56)	
Patient race				
Black	2239 (18)	9055 (11)	11 294 (12)	
White	8153 (64)	44 754 (52)	52 907 (54)	<.001
Other^a^	1972 (16)	4390 (5)	6362 (6)	
Missing	335 (3)	27 252 (32)	27 587 (28)	
Rank				
Enlisted junior	218 (2)	2221 (3)	2439 (2)	<.001
Enlisted senior	6215 (49)	60 607 (71)	66 822 (68)	
Junior officer	474 (4)	7693 (9)	8167 (8)	
Senior officer	2483 (20)	11 457 (13)	13 940 (14)	
Other	3249 (26)	3473 (4)	6722 (7)	
Missing	60 (<1)	0	60 (0.06)	
CCI total				
None	12 065 (95)	72 663 (85)	84 728 (86)	<.001
1	572 (5)	10 422 (12)	10 994 (11)	
2	59 (<1)	1965 (2)	2024 (2)	
≥3	3 (<1)	401 (<1)	404 (<1)	
Region of MTF				
Midwest	831 (7)	13 481 (16)	14 312 (15)	<.001
Northeast	219 (2)	5396 (6)	5615 (6)	
Other	738 (6)	231 (0.27)	969 (1)	
South	6962 (55)	52 181 (61)	59 143 (60)	
West	3880 (31)	14 162 (17)	18 042 (18)	
Inactive MTFs	69 (<1)	0	69 (<1)	
Beneficiary status				
Active duty	5653 (45)	12 540 (15)	18 193 (19)	<.001
Retired	3665 (29)	35 318 (41)	38 983 (40)	
Dependent	2747 (22)	37 217 (44)	39 964 (41)	
Other	632 (5)	53 (<1)	685 (<1)	
Unknown	2 (<1)	323 (<1)	325 (<1)	
Age category				
Youth (10-24 y)	82 (<1)	158 (<1)	240 (<1)	<.001
Young adult (25-44 y)	3364 (26)	9816 (11)	13 180 (13)	
Middle adult (45-64 y)	8507 (67)	75 079 (88)	83 586 (85)	
Older adult (≥65)	746 (6)	398 (<1)	1144 (1)	

Abbreviations: CCI, Charlson Comorbidity Index; MTF, military treatment facility; NA, not appliable.

^a^ Including American Indian or Alaskan Native, Asian or Pacific Islander, and other race.

**Table 2.** Association of Care Setting With the Provision of Low-Value Surgery

Variable	OR (95% CI)	*P* value
Care setting		
Direct	1 [Reference]	NA
Private	1.41 (1.38-1.45)	<.001
Time period		
2016-2019	1 [Reference]	NA
2020-2023	0.85 (0.83-0.87)	<.001
Sex		
Women	1 [Reference]	NA
Men	0.87 (0.86-0.89)	<.001
Race		
Black	1.10 (1.07-1.12)	<.001
White	1 [Reference]	NA
Other^a^	0.96 (0.94-0.99)	.027
Rank		
Senior officer	1 [Reference]	NA
Enlisted junior	0.52 (0.49-0.55)	<.001
Enlisted senior	0.87 (0.85-0.89)	<.001
Junior officer	0.83 (0.80-0.86)	<.001
Other	0.90 (0.87-0.94)	<.001
Comorbidities		
None	1 [Reference]	NA
1	1.00 (0.98-1.03)	.62
2	1.05 (0.99-1.12)	.092
≥3	0.78 (0.68-0.90)	<.001
Census region		
South	1 [Reference]	NA
Midwest	1.26 (1.23-1.29)	<.001
Northeast	1.23 (1.18-1.28)	<.001
Other	1.06 (0.98-1.14)	.14
West	0.90 (0.88-0.92)	<.001
Age, per 1-y increase	1.07 (1.06-1.07)	<.001

Abbreviations: NA, not applicable; OR, odds ratio.

^a^ Including American Indian or Alaskan Native, Asian or Pacific Islander, and other race.

Interaction analysis found that odds of low-value care were significantly lower in each respective sector for 2020 to 2023 vs 2016 to 2019 (direct care: OR, 0.78; 95% CI, 0.73-0.83; *P* < .001; private sector: OR, 0.93; 95% CI, 0.91-0.96; *P* < .001) (Table 3). However, procedures from the private sector had significantly greater odds of low-value care compared with direct care in each time period (2016-2019: OR, 1.29; 95% CI, 1.24-1.35; *P* < .001; 2020-2023: OR, 1.55; 95% CI, 1.47-1.63; *P* < .001).

**Table 3.** Estimations of the Association of the Interaction Between Care Setting and Time Period From the Primary Regression Analysis

Environment of care and time period	OR (95% CI)	*P* value
Direct 2020-2023 vs direct 2016-2019	0.78 (0.73-0.83)	<.001
Private 2016-2019 vs direct 2016-2019	1.29 (1.24-1.35)	<.001
Private 2020-2023 vs direct 2016-2019	1.21(1.16-1.26)	<.001
Private 2020-2023 vs private 2016-2019	0.93 (0.91-0.96)	<.001
Private 2020-2023 vs direct 2020-2023	1.55 (1.47-1.63)	<.001
Private 2016-2019 vs direct 2020-2023	1.65 (1.57-1.74)	<.001

Abbreviation: OR, odds ratio.

In secondary testing, when limited to non–active-duty personnel, the unadjusted rates of low-value care within each procedure remained higher in the private sector for most categories (Table 4). After adjusting for case mix, low-value ankle arthroscopy (OR, 0.60; 95% CI, 0.46-0.77; *P* < .001) and low-value partial meniscectomy (OR, 0.55; 95% CI, 0.51-0.59; *P* < .001) were both significantly less likely in the private sector.

**Table 4.** Models Assessing the Association Between Care Setting and Provision of Low-Value Surgical Care by Procedure

Procedure and care setting	Procedures, No. (%)		Unadjusted		Adjusted	
	Low-value	Non–low value	OR (95% CI)	*P* value	OR (95% CI)	*P* value
Acromioplasty						
Direct care	943 (16)	4781 (84)	1 [Reference]	NA	1 [Reference]	NA
Private sector	8434 (24)	26 194 (76)	1.73 (1.60-1.88)	<.001	1.70 (1.57-1.85)	<.001
Rotator cuff repair						
Direct care	1278 (19)	5309 (81)	1 [Reference]	NA	1 [Reference]	NA
Private sector	9528 (33)	19 444 (67)	2.09 (1.96-2.24)	<.001	2.03 (1.89-2.19)	<.001
Partial meniscectomy						
Direct care	4142 (60%)	2706 (40)	1 [Reference]	NA	1 [Reference]	NA
Private sector	26 632 (61)	16 851 (39)	1.02 (0.97-1.08)	.34	0.55 (0.51-0.59)	<.001
Ankle arthroscopy						
Direct care	183 (26)	533 (74)	1 [Reference]	NA	1 [Reference]	NA
Private sector	508 (21)	1967 (79)	0.81 (0.65-0.99)	.05	0.60 (0.46-0.77)	<.001
Wrist arthroscopy						
Direct care	74 (28)	192 (72)	1 [Reference]	NA	1 [Reference]	NA
Private sector	401 (29)	993 (71)	1.01 (0.75-1.37)	.90	0.73 (0.50-1.06)	.10

Abbreviation: OR, odds ratio.

## Discussion

To our knowledge, this cohort study is the first to examine the association of reimbursement policy with the provision of low-value surgical care among procedures with a broad translational capacity in the current US health care environment. A strength of our investigation is our large sample with comprehensive surveillance of beneficiaries in the time period under investigation, the 2-pronged nature of the MHS incorporating mutually exclusive salaried and fee-for-service networks, and a patient population that is representative of the US population aged 64 years or younger. We also used literature-based definitions specific to low-value care^1,15,16,17,18,19,20,21,22,23,24,25^ rather than relying on rates of surgical interventions within the different environments of care as a proxy. We found that low-value surgical care was significantly more likely to occur in the private sector than in the direct care setting overall and when separately considering the time periods 2016 to 2019 vs 2020 to 2023.

In contrast to previous studies involving low-value care, the procedures we included are universally performed in most health care settings, are elective, and are associated with quality of life and physical function and performance, as opposed to limb- or life-threatening conditions. As a result, these interventions may possess the greatest potential for clinician-induced demand. Many of these procedures have been shown to have a volume-based performance divorced from surgeon density,^11^ a potential indicator of practice variation associated with clinician-induced demand. Furthermore, the procedures we selected have broad translational capacity to other elective procedures with similar potential for clinician-induced demand in other disciplines, including coronary revascularization, interventional radiology, carotid artery procedures, and women’s health.^11,12,14^

Drivers of low-value care are thought to include financial incentives, patient and clinician knowledge, bias and clinical uncertainty, and power dynamics within human relationships,^3,5,7^ with prominent intersections in the concept of clinician-induced demand.^6^ Clinician-induced demand may also be influenced through organizational efforts focused on maintaining procedural volume and competencies, preserving surgical block allocations, or the Lake Wobegon Effect, where surgeons come to believe that otherwise low-value interventions exhibit superior results in their hands.^29^

We believe that the results of our investigation are important for surgeons, health care administrators, third-party payers, and the federal government, irrespective of affiliation with the MHS or TRICARE. The most important finding is that changing a reimbursement model from fee-for-service to salaried may be associated with a 41% difference in the odds of low-value surgical intervention. This is aligns with previous work that found a higher likelihood of surgical intervention as opposed to nonoperative treatment for carotid stenosis^6^ and the use of more intense and expensive surgical interventions for spinal conditions in fee-for-service vs salaried health care networks.^4^ Altering the reimbursement model could potentially address financial motivations and certain implicit biases that may manifest in association with this domain.

However, it is clear from our analysis that such a move alone is unlikely to eliminate low-value surgical services in their entirety. Foremost, the salaried direct care system exhibited a 20% rate of low-value surgery, although significant reductions of approximately 22% lower odds were observed within this environment between the 2016 to 2019 and 2020 to 2023 periods. In the same time frame, there was only a 7% reduction in the odds of low-value care in the private sector. This would suggest that the administrative changes in the MHS over this time window, such as centralized consolidation under the auspices of the Defense Health Agency^14^ and the cumulative effect of more than a decade of concerted effort at reducing low-value care within the MHS as a whole,^2^ are exerting a positive impact. However, the provision of low-value surgery was not uniform across all interventions, and, in our secondary tests using case-mix adjustment, we found that low-value partial meniscectomy and ankle arthroscopy were more likely in direct care. This represents an opportunity for improvement and an avenue of further investigation as we seek to understand the reasons why these procedures are outliers in direct care when it comes to provision of low-value surgery. Approaches, such as the intentional evaluation of the determinants of low-value surgical care, quality collaboratives that target and monitor the provision of low-value surgical services, and clinician decision support and dashboards that provide feedback on the performance of low-value surgical care,^29^ are relevant to both direct care and the private sector setting in the MHS and scalable to civilian health care networks on the whole.

Accountable care organizations (ACOs) and bundled payments were both previously viewed as effective strategies against low-value care, although neither have been found to perform well in this regard.^30,31,32,33,34^ Bundled models^30,31^ may influence preoperative testing or the use of postprocedural services but do not necessarily disincentivize the performance of low-value procedures in and of themselves. A 2019 study by Modi et al^32^ reported that the formation of ACOs was not associated with reduced frequency of low-value procedures, like arthroscopic partial meniscectomy. Schoenfeld et al^33^ found that the implementation of ACOs was not associated with changes in the use of high-intensity interventions in the field of spine surgery, while Hollingsworth et al^34^ noted similar findings for coronary revascularization. We believe that this illustrates that low-value surgery is unlikely to be passively impacted by health reform efforts intended for other purposes and directed interventions specifically designed to address the provision of low-value surgery are necessary. Our results would support initiatives targeting partial meniscectomy and ankle arthroscopy in the MHS direct care setting, while also emphasizing a focus on these procedures in addition to acromioplasty, rotator cuff repair, and wrist arthroscopy in the civilian sector and the US health care system on the whole.

### Limitations

This study has some limitations. This is a retrospective study that relies on health care claims. As a result, we do not possess information on the severity of disease, clinical rationale, or preoperative evaluations and discussions that went into the decision to perform surgery. However, we emphasize that our characterization of low-value surgery is based on evidence provided in the literature, and these factors are unlikely to alter the characterization of low-value care at the network level with the health policy lens we have used here. Second, our determinations regarding low-value care are based on coding algorithms, although, again, these are informed by other studies and existing publications in the literature. As such, we maintain this embodies an effective definition of low-value surgery from the health policy standpoint. Third, provision of surgical care for active-duty personnel has both personal health and military readiness components^26,35^ that do not always translate to individuals without a military affiliation. In some situations, the decision for surgery may be driven by short-term needs and a realistic appreciation of postsurgical implications on the part of patient and clinician^35^; yet surgery is still selected as the only viable means of extending a military career or facilitating a servicemember’s reaching an important milestone, such as retirement. To address this fact, we performed secondary analyses in which we limited consideration to procedures performed on individuals who were not involved with active duty. Additionally, while we were able to characterize changes in low-value surgery over time and between health care environments within the MHS, we are unable to definitively speak to the behaviors, approaches, and policies that underpin the differences we observed. We are also unable to speak to other approaches that may reduce low-value care in the direct care or private sector. These are areas of future investigation for which we envision using mixed-methods techniques.

## Conclusions

In this cohort study, we found significant differences in the provision of low-value surgical care for common elective procedures between the salaried, direct care setting and the fee-for-service private sector. While secular trends in the reduction of low-value surgery were appreciated across both health care environments between 2016 to 2019 and 2020 to 2023, direct care maintained a significantly lower likelihood of low-value care in both time periods. We believe that this supports the contention that changing clinician reimbursement models from fee-for-service to salaried may result in a lower rate of low-value surgical care. Changing the reimbursement model from fee-for-service to salaried may be associated with as much as a 41% change in the odds of low-value surgical intervention.

## Notes

Supplement 1. **eTable.** Sociodemographic and clinical characteristics of the population under study stratified by low-value care definition **eAppendix.** Technical appendix

Supplement 2.
Data Sharing Statement

## References

[1] Ponkilainen V, Laurema A, Mattila VM, Karjalainen T. Regional variation in low-value musculoskeletal surgery: a nationwide study from the Finnish Care Register. Acta Orthop. 2024;95:553-561. 39301978 10.2340/17453674.2024.41930 PMC11415780

[2] Koehlmoos TP, Madsen CK, Banaag A, Haider AH, Schoenfeld AJ, Weissman JS. Assessing low-value health care services in the Military Health System. Health Aff (Millwood). 2019;38(8):1351-1357. doi:10.1377/hlthaff.2019.00252 31381388

[3] Birkmeyer JD, Reames BN, McCulloch P, Carr AJ, Campbell WB, Wennberg JE. Understanding of regional variation in the use of surgery. Lancet. 2013;382(9898):1121-1129. doi:10.1016/S0140-6736(13)61215-5 24075052 PMC4211114

[4] Lawlor MC, Cirillo MN, Holly KE, . Is civilian hospital treatment of lumbar spinal disorders associated with greater odds of fusion procedures? Clin Orthop Relat Res. 2025;483(10):1939-1947. doi:10.1097/CORR.0000000000003487 40153716 PMC12453319

[5] Shrank WH, Rogstad TL, Parekh N. Waste in the US health care system: estimated costs and potential for savings. JAMA. 2019;322(15):1501-1509. doi:10.1001/jama.2019.13978 31589283

[6] Nguyen LL, Smith AD, Scully RE, . Provider-induced demand in the treatment of carotid artery stenosis: variation in treatment decisions between private sector fee-for-service vs salary-based military physicians. JAMA Surg. 2017;152(6):565-572. doi:10.1001/jamasurg.2017.0077 28249083 PMC5831423

[7] Saini V, Garcia-Armesto S, Klemperer D, . Drivers of poor medical care. Lancet. 2017;390(10090):178-190. doi:10.1016/S0140-6736(16)30947-3 28077235

[8] Schoenfeld AJ, Kaji AH, Haider AH. Practical guide to surgical data sets: military health system Tricare encounter data. JAMA Surg. 2018;153(7):679-680. doi:10.1001/jamasurg.2018.0480 29617526

[9] Gimbel RW, Pangaro L, Barbour G. America’s “undiscovered” laboratory for health services research. Med Care. 2010;48(8):751-756. doi:10.1097/MLR.0b013e3181e35be8 20613659

[10] Schoenfeld AJ, Jiang W, Harris MB, . Association between race and postoperative outcomes in a universally insured population versus patients in the State of California. Ann Surg. 2017;266(2):267-273. doi:10.1097/SLA.0000000000001958 27501169

[11] Jain NB, Peterson E, Ayers GD, Song A, Kuhn JE. US geographical variation in rates of shoulder and knee arthroscopy and association with orthopedist density. JAMA Netw Open. 2019;2(12):e1917315. doi:10.1001/jamanetworkopen.2019.17315 31825507 PMC6991208

[12] Chaudhary MA, Bhulani N, de Jager EC, . Development and validation of a bedside risk assessment for sustained prescription opioid use after surgery. JAMA Netw Open. 2019;2(7):e196673. doi:10.1001/jamanetworkopen.2019.6673 31290987 PMC6624809

[13] Scully RE, Schoenfeld AJ, Jiang W, . Defining optimal length of opioid pain medication prescription after common surgical procedures. JAMA Surg. 2018;153(1):37-43. doi:10.1001/jamasurg.2017.3132 28973092 PMC5833616

[14] Schoenfeld AJ, Cooper Z, Banaag A, . Long-term prescription opioid use following surgery in the US (2017-2022): a population-based study. Lancet Reg Health Am. 2024;40:100948. doi:10.1016/j.lana.2024.100948 39763498 PMC11703578

[15] Geurkink TH, van Bodegom-Vos L, Nagels J, . The relationship between publication of high-quality evidence and changes in the volume and trend of subacromial decompression surgery for patients with subacromial pain syndrome in hospitals across Australia, Europe and the United States: a controlled interrupted time series analysis. BMC Musculoskelet Disord. 2023;24(1):456. doi:10.1186/s12891-023-06577-6 37270498 PMC10239046

[16] Paavola M, Malmivaara A, Taimela S, ; Finnish Subacromial Impingement Arthroscopy Controlled Trial (FIMPACT) Investigators. Subacromial decompression versus diagnostic arthroscopy for shoulder impingement: randomised, placebo surgery controlled clinical trial. BMJ. 2018;362:k2860. doi:10.1136/bmj.k2860 30026230 PMC6052435

[17] Paloneva J, Lepola V, Karppinen J, Ylinen J, Äärimaa V, Mattila VM. Declining incidence of acromioplasty in Finland. Acta Orthop. 2015;86(2):220-224. doi:10.3109/17453674.2014.977703 25340548 PMC4404774

[18] Blom AW, Donovan RL, Beswick AD, Whitehouse MR, Kunutsor SK. Common elective orthopaedic procedures and their clinical effectiveness: umbrella review of level 1 evidence. BMJ. 2021;374(1511):n1511. doi:10.1136/bmj.n1511 34233885 PMC8262448

[19] Karjalainen TV, Jain NB, Heikkinen J, Johnston RV, Page CM, Buchbinder R. Surgery for rotator cuff tears. Cochrane Database Syst Rev. 2019;12(12):CD013502. doi:10.1002/14651858.CD013502 31813166 PMC6900168

[20] Karjalainen VL, Harris IA, Räisänen M, Karjalainen T. Minimal invasions: is wrist arthroscopy supported by evidence—a systematic review and meta-analysis. Acta Orthop. 2023;94:200-206. doi:10.2340/17453674.2023.11957 37114362 PMC10141317

[21] Katz JN, Brophy RH, Chaisson CE, . Surgery versus physical therapy for a meniscal tear and osteoarthritis. N Engl J Med. 2013;368(18):1675-1684. doi:10.1056/NEJMoa1301408 23506518 PMC3690119

[22] Kise NJ, Risberg MA, Stensrud S, Ranstam J, Engebretsen L, Roos EM. Exercise therapy versus arthroscopic partial meniscectomy for degenerative meniscal tear in middle aged patients: randomised controlled trial with two year follow-up. BMJ. 2016;354:i3740. doi:10.1136/bmj.i3740 27440192 PMC4957588

[23] Kukkonen J, Joukainen A, Lehtinen J, . Treatment of nontraumatic rotator cuff tears: a randomized controlled trial with two years of clinical and imaging follow-up. J Bone Joint Surg Am. 2015;97(21):1729-1737. doi:10.2106/JBJS.N.01051 26537160

[24] Sihvonen R, Paavola M, Malmivaara A, ; Finnish Degenerative Meniscal Lesion Study (FIDELITY) Group. Arthroscopic partial meniscectomy versus sham surgery for a degenerative meniscal tear. N Engl J Med. 2013;369(26):2515-2524. doi:10.1056/NEJMoa1305189 24369076

[25] Glazebrook MA, Ganapathy V, Bridge MA, Stone JW, Allard JP. Evidence-based indications for ankle arthroscopy. Arthroscopy. 2009;25(12):1478-1490. doi:10.1016/j.arthro.2009.05.001 19962076

[26] Patzkowski JC, Rivera JC, Ficke JR, Wenke JC. The changing face of disability in the US Army: the Operation Enduring Freedom and Operation Iraqi Freedom effect. J Am Acad Orthop Surg. 2012;20(suppl 1):S23-S30. doi:10.5435/JAAOS-20-08-S23 22865131

[27] Deyo RA, Cherkin DC, Ciol MA. Adapting a clinical comorbidity index for use with *ICD-9-CM* administrative databases. J Clin Epidemiol. 1992;45(6):613-619. doi:10.1016/0895-4356(92)90133-8 1607900

[28] Henry AJ, Hevelone ND, Lipsitz S, Nguyen LL. Comparative methods for handling missing data in large databases. J Vasc Surg. 2013;58(5):1353-1359.e6. doi:10.1016/j.jvs.2013.05.008 23830314

[29] Berlin NL, Skolarus TA, Kerr EA, Dossett LA. Too much surgery: overcoming barriers to deimplementation of low-value surgery. Ann Surg. 2020;271(6):1020-1022. doi:10.1097/SLA.0000000000003792 32209904 PMC7269189

[30] Berlin NL, Kamdar N, Syrjamaki J, Sears ED. Health-care patterns for three common elective surgeries: Implications for bundled payment models. J Surg Res. 2023;291:414-422. doi:10.1016/j.jss.2023.06.028 37517349

[31] Rana AJ, Bozic KJ. Bundled payments in orthopaedics. Clin Orthop Relat Res. 2015;473(2):422-425. doi:10.1007/s11999-014-3520-2 24554458 PMC4294917

[32] Modi PK, Kaufman SR, Borza T, . Medicare accountable care organizations and use of potentially low-value procedures. Surg Innov. 2019;26(2):227-233. doi:10.1177/1553350618816594 30497340 PMC6503656

[33] Schoenfeld AJ, Sturgeon DJ, Harris MB, . Changes in the use of lumbar arthrodesis procedures within accountable care organizations. Spine (Phila Pa 1976). 2019;44(7):488-493. doi:10.1097/BRS.0000000000002862 30234797

[34] Hollingsworth JM, Nallamothu BK, Yan P, . Medicare accountable care organizations are not associated with reductions in the use of low-value coronary revascularization. Circ Cardiovasc Qual Outcomes. 2018;11(6):e004492. doi:10.1161/CIRCOUTCOMES.117.004492 29903936 PMC6005663

[35] Schoenfeld AJ. Low back pain in the uniformed service member: approach to surgical treatment based on a review of the literature. Mil Med. 2011;176(5):544-551. doi:10.7205/MILMED-D-10-00397 21634300

