# Supplementary material for: Surgical Low-Value Care Between Fee-For-Service and Salaried Health Care Systems
Source: JAMA Netw Open. 2025 Dec 2;8(12):e2546213. doi: 10.1001/jamanetworkopen.2025.46213 (PMC12673412; doi:10.1001/jamanetworkopen.2025.46213)
Supplement: Supplement 1. — eTable. Sociodemographic and clinical characteristics of the population under study stratified by low-value care definition eAppendix. Technical appendix [file jamanetwopen-e2546213-s001.pdf]

Supplemental Online Content

Schoenfeld AJ, Holly KE, Cirillo MN, et al. Surgical low-value care between fee-for-service and salaried health care systems. *JAMA Netw Open*. 2025;8(12):e2546213. doi:10.1001/jamanetworkopen.2025.46213

eTable. Sociodemographic and clinical characteristics of the population under study stratified by low-value care definition

eAppendix. Technical appendix

This supplemental material has been provided by the authors to give readers additional information about their work.

© 2025 Schoenfeld AJ et al. *JAMA Network Open*.

eTable 1. Sociodemographic and clinical characteristics of the population under study stratified by low-value care definition.

	Low Value Care (n=98,150, 32%)	Procedures performed alone but not low value care (n=32,180, 11%)	Procedures performed in conjunction with other surgical procedural interventions (n=174,578, 57%)	Total (n=304,908)	P Value
**Care Setting**					
Private	85,451 (35)	20,267 (8)	136,282 (56)	242,000 (79)	
Direct	12,699 (20)	11,913 (19)	38,296 (61)	62,908 (21)	<0.001
**Time Period**					
2016-2019	54,553 (32)	17,901 (11)	95,737 (57)	168,191 (55)	
2020-2023	43,597 (32)	14,279 (10)	78,841 (58)	136,717 (45)	<0.001
**Type of Surgery**					
Acromioplasty	19,485 (22)	21(0.02)	68,215 (78)	87,721 (29)	
Ankle Arthroscopy	1,542 (11)	3,379 (23)	9,568 (66)	14,489 (5)	
Partial Meniscectomy	56,928 (44)	23,434 (18)	48,001 (38)	128,363 (42)	
Rotator cuff repair	19,093 (28)	2,665 (4)	45,529 (68)	67,287 (22)	
Wrist Arthroscopy	1,102 (16)	2,681 (38)	3,265 (46)	7,048 (2)	<0.001
**Care Setting & Time Period Interaction**					
Direct 2016-2019	8,030 (21)	7,088 (19)	22,381 (60)	37,499 (12)	
Direct 2020-2023	4,669 (18)	4,825 (19)	15,915 (63)	25,409 (8)	
Private 2016-2019	46,523 (36)	10,813 (8)	73,356 (56)	130,692 (43)	
Private 2020-2023	38,928 (35)	9,454 (8)	62,926 (57)	111,308 (37)	<0.001
**Gender**					
Women	43,237 (38)	8,194 (7)	63,829 (55)	115,260 (38)	
Men	54,913 (29)	23,986 (13)	110,749 (58)	189,648 (62)	<0.001
**Race**					
White	52,907 (31)	19,531 (11)	99,953 (58)	172,391 (57)	
Black	11,294 (30)	4,424 (12)	21,409 (58)	37,127 (12)	
Other [Asian/ Pacific Islander, American Indian/Alaskan Native and Other Race]	6,362 (25)	3,404 (14)	15,245 (61)	25,011 (8)	
Missing	27,587 (39)	4,821 (7)	37,971 (54)	70,379 (23)	<0.001
**Rank**					
Enlisted Junior	2,439 (11)	7,028 (31)	13,321 (59)	22,788 (7)	
Enlisted Senior	66,822 (34)	18,279 (9)	113,709 (57)	198,810 (65)	
Junior Officer	8,167 (30)	3,656 (14)	15,562 (57)	27,385 (9)	
Senior Officer	13,940 (40)	1,248 (4)	19,612 (57)	34,800 (11)	
Other	6,722 (32)	1,962 (9)	12,208 (58)	20,892 (7)	
Missing	60 (26)	7 (3)	166 (71)	233 (0.08)	<0.001
**Comorbidities**					
None	84,728 (31)	31,251 (11)	157,317 (58)	273,296 (90)	
1	10,994 (42)	891 (3)	14,376 (55)	26,261 (9)	
2	2,024 (46)	35 (0.79)	2,374 (54)	4,433 (1)	
3 or more	404 (44)	3 (0.33)	511 (56)	918 (0.30)	<0.001
**Hospital Region**					
Midwest	14,312 (38)	3,726 (10)	19,416 (52)	37,454 (12)	
Northeast	5,615 (35)	1,902 (12)	8,616 (53)	16,133 (5)	
Other	969 (19)	1,243 (24)	3,011 (58)	5,223 (2)	
South	59,143 (33)	16,430 (9)	103,567 (58)	179,140 (59)	
West	18,042 (27)	8,770 (13)	39,652 (60)	66,464 (22)	
Unknown	69 (14)	109 (22)	316 (64)	494 (0.16)	<0.001
**Beneficiary Status**					
Active Duty	18,193 (18)	22,954 (22)	61,346 (60)	102,493 (34)	
Retired	38,983 (42)	1,088 (1)	53,103 (57)	93,174 (31)	

© 2025 Schoenfeld AJ et al. *JAMA Network Open*.

Dependent	39,964 (38)	7,809 (7)	58,021 (55)	105,794 (35)	
Other	685 (31)	139 (6)	1,424 (63)	2,248 (0.74)	
Unknown	325 (27)	190 (16)	684 (57)	1,199 (0.39)	<0.001
**Age Category**					
Youth (10-24)	240 (1)	8,530 (39)	13,195 (60)	21,965 (7)	<0.001
Young Adult (25-44)	13,180 (14)	23,650 (26)	55,069 (60)	91,899 (30)	
Middle Adult (45-64)	83,586 (44)	0 (0)	104,525 (56)	188,111 (62)	
Elderly (65+)	1,144 (39)	0 (0)	1,789 (61)	2,933 (0.96)	

© 2025 Schoenfeld AJ et al. *JAMA Network Open*.

Technical Appendix

Inclusion:

Patients who underwent acromioplasty; patients who underwent partial meniscectomy; patients who underwent wrist arthroscopic debridement; patients who underwent ankle arthroscopy; patients who underwent rotator cuff repair defined using the coding algorithm below.

Exclusion:

Procedures associated with infection (including sepsis), tumor (including metastases), or trauma to the shoulder, knee, wrist or ankle defined using the codes below.

Surgery/NOMESCO code	CPT Code
Acromioplasty<br>**NBG10**, Acromioplasty<br>**NBG15** Acromioplasty, arthroscopic	23130, 29826
Partial Meniscectomy<br>**NGD05** partial excision of meniscus of knee, arthroscopic	29880, 29881
Wrist Arthroscopy<br>**NDF25** Operation of osteochondritis of joint of wrist, arthroscopic	29843, 29844, 29845, 29846, 29847
Ankle Arthroscopy<br>**NHA30** Exploration of joint of ankle or foot, arthroscopic<br>**NHF*** Operations on synovia and joint surfaces of ankle and foot	29891, 29894, 29897, 29898
Rotator Cuff Repair<br>**NBL00** Suture or reinsertion of rotator cuff<br>**NBL05** Arthroscopic suture or reinsertion of rotator cuff	29827, 23410, 23412, 23420

Exclusion Criteria ICD-10-CM Codes:

General

Infection: A17.81, A17.89, A18.01, A40.1, A41.01, A41.02, A41.9, B20, A49.1, T86.832, T81.42, T81.42XA, T81.42XD, T81.42XS, L08.9, A02.23, A18.02, A39.83, A39.84, A54.40, A54.42, A54.43, A54.49, M00.00, M00.09, M00.10, M00.19, M00.20, M00.29, M00.09, M00.10, M00.80, M00.89, M01.X0, M01.X9, M00.9, T81.40XA, T81.40XD, T81.40XS, T81.41XA, T81.42XS, T81.49XA, T81.49XS

Trauma: S06.4X0A, S06.4X9A, S06.6X0A, S12.000A, S12.000K, S12.030A, S12.100A, S12.100K, S12.101A, S12.110A, S12.110K, S12.111A, S12.111K, S12.120, S12.120K, S12.190A, S12.190G, S12.190K, S12.2, S12.200A, S12.300A, S12.300K, S12.400, S12.400A, S12.401A, S12.500A, S12.500B, S12.501A, S12.501G, S12.530, S12.590A, S12.600A, S12.690G, S12.691A, S12.9XXA, S12.9XXD, S13.0XXA, S13.120A, S13.121A, S13.140A, S13.141A, S13.150A, S13.151A, S13.160A, S13.161A, S13.171A, S14.102, S14.103A, S14.104A, S14.105A, S14.106A, S14.109A, S14.112A, S14.115A, S14.121A, S14.123A, S14.123S, S14.124A, S14.125A, S14.127A, S14.129A, S14.155, S14.155A, S14.156A,

© 2025 Schoenfeld AJ et al. *JAMA Network Open*.

S14.2XXA, S22.001A, S22.009A, S22.010A, S22.060A, S22.068A, S22.068D, S22.069A, S22.070, S22.070A, S22.079A, S22.081A, S22.081B, S22.082A, S22.082B, S22.088A, S22.089A, S22.089D, S23.150A, S23.161A, S24.101, S24.104A, S24.151A, S24.159A, S24.2XXS, S32.001, S32.008K, S32.009A, S32.009K, S32.009S, S32.01, S32.011, S32.011A, S32.012A, S32.018A, S32.019A, S32.021A, S32.022A, S32.028A, S32.029A, S32.031A, S32.032A, S32.041A, S32.048A, S32.049A, S32.051A, S32.10XA, S33.0, S33.0XXA, S33.0XXD, S33.1, S33.121S, S33.141A, S33.39XA, S33.5, S34.21XA, T79..9XXD, Z87.82 , M87.20

Tumor: C06.1, C34.12, C34.90, C41, C41.2, C41.9 C47.3, C47.9, C49.6, C50.919, C54.1, C63.1, C64.2, C64.9, C70.1, C71.1, C71.8, C72.0, C79.31, C79.49, C79.5, C79.51, C79.52, C79.89, C7B.03, C85.89, C90.00, C90.02, C90.30, D16.6, D17.79, D18.09, D32.1, D32.9, D33.1, D33.4, D33.9, D35.2, D36.1, D36.10, D36.11, D36.14, D36.17, D42.1, D43.4, D47.Z9, D48.0, D49.2, D49.7A , C72.1, C72, C49.8, C49.9, C40.80, C40.81,C40.82, C40.90, M84.58XA

Meniscectomy

Infection: M71.162, M71.162, S80.211A, S80.212A, S80.221A, S80.222A, M01.X6, M01.X61, M01.X62, M01X69, S80.251A, S80.252A,S80.261A, S80.262A, L08.9, S81, A54.42, A69.23, A54.42, M00.069, M00.162, M00.169, M00.062, M00.061, M00.161, M71.061, M71.062, M00.269, M00.262, M00.861, M00.862, M71.169

Trauma: S72.145A, S88.11, S88.011A, S88.012A, S88.021A, S88.022A, S88.011D, S88.011S, S88.012D, S88.012S, S88.019A, S88.019D, S88.019S, S88.021D, S88.021S, S88.022D, S88.022S, S88.029, S88.029A, S88.029D, S88.029S, S88.121A, S88.122A, S88.111A, S88.112A, S83, M24.661, M24.662, M87.251, M87.252 M87.256, M87.263, M87.266, S81.05, M23.51, M23.53, M24.461, M24.462, M87.251, M87.252, M87.29, S81.009A, S83.91XA, S83.92XA, S83.101A, S83.102A, M23.8X9, M24.469, M23.50, S87.01XA, S87.02XA, M24.361, M24.362, M24.369, S83.104A, S83.105A, S83.103A, S83.103D, S83.106A, S83.106D, S83.113, S83.116, S83.123, S83.126, S83.133, S83.136, S83.401S, S83.402S, S83.409, S83.409S, S83.419S, S83.429S, S83.501S, S83.502S, S83.509S, S83.519S, S83.529S, S83.60, S83.61, S83.62, S83.90XD, S83.91XD S83.143, S83.146, S81.011A, S81.012A, M23.601, M23.602, M23.609, M23.61, M23.62, M23.63, M23.64, M23.671, M23.672, M67.879, T79.A21A, T79.A21S, T79.A22A, T79.A22S, T79.A29A, T79.A29S, S84.00XA, S84.00XS, S84.01XA, S84.01XS, S84.02XA, S84.02XS, S84.10XA, S84.10XS, S84.11XA, S84.11XS, S84.12XA, S84.12XS, S84.20XA, S84.20XS, S84.21XA, S84.21XS, S84.22XA, S84.22XS, S84.801A, S84.801S, S84.802A, S84.802S, S84.809A, S84.809S, S84.90XA, S84.90XS, S84.91XA, S84.91XS, S84.92XA, S84.92XS, S85, S86

Tumor: C49.2, C49.21, C49.22, C40.20, C40.21, C,40.22, C40.30, C40.31, C40.32, C40.82, C40.91, C40.92, C47.20, C47.21, C47.22, C47.8, M84.551A, M84.552A, M84.553A, M84.561A, M84.562A, M84.563A, M84.564A, M84.569A, D16.3, D16.2, M84.561S, M84.562S, M84.563S, M84.564S, M84.569S

© 2025 Schoenfeld AJ et al. *JAMA Network Open*.

Rotator Cuff/ Acromioplasty

Infection: M01.X1, M01.X19, M00.011, M00.019, M00.012, M00.111, M00.112, M00.119, M00.211, M00.212, M00.219, M00.811, M00.812, M00.819, M01.X11, M00.X12, M60.01, M60.011, M60.012, M60.019, L08.9, M71.012, M71.011, M71.019, M86.211, M86.212, M86.219, M65.011, M65.012, M86.619, M86.019, M86.319, M86.119, M89.611, M89.612, M89.619, M86.519, M86.111, M86.112, M86.411, M86.011, M86.012, M86.311, M86.312

Trauma: M24.411, M24.412, M25.211, M25.212, M12.511, M12.512, M12.519, M24.419, M19.111, M19.112, M19.119, S42.9, M61.011, M61.012, M61.019, S43.90, M25.311, M25.312, M86.219, S43.001A, S43.002A, M87.21, M87.221, M87.222, M87.219, S43.401A, S41.009A, S43.30, S41.051A, S41.052A, S41.059A, S43.004A, S41.011A, S41.012A, S42.91XA, S42.92XA, S48.011S, S48.012S, S48.019S, S48.029S, S48.9, S48.029A, S48.021A, S48.022A, S42, S41, S43.90, S43.90, S42.03, S42.002A, S42.009S, S42.001A, S42.002B, S42.021A, S42.021B, S42.022A, S42.022B, S42.013, S42.001K, S42.001P, S42.002K, S42.002P, S42.009A, S42.009B, S42.031A, S42.031B, S42.032A, S42.032B, S42.012A, S42.011A, S42.011B, S42.012B, S42.014, S42.016, S46.017S, S42.018S, S42.019S, S42.026S, S42.033S, S42.034S, S42.035S, S42.036S, S42.017A, S42.034A, S42.017B S42.018A, S42.018B, S42.019A, S42, S42.024A, S42.026A, S42.026B, S42.033A, S42.033B, S42.035A, S42.035B, S42.013A, S42.013S, M84.311A, M84.312A, M84.411A, M84.412A, S42.101A, S42.101B, S42.101S, S42.102A, S42.102B, S42.102S S42.111A, S42.111B, S42.111S, S42.112A, S42.112B, S42.112S, S42.109A, S42.109B, S42.109S, S42.199A, S42.199B, S42.199S S42.191A, S42.191B, S42.191S, S42.192A, S42.192B, S42.192S, S42.1, S42.0, S42.2, S42.3, S47.1XXA, S47.1XXS, S47.2XXA, S47.2XXS, S47.XXA, S47.9XXS, S44, S45, S46

Tumor: M84.511A, M84.511S, M84.512S M84.512A, C41.3, C40.0, C40.1, D16.0, D16.1, C49.1, D36.12

Wrist Arthroscopy

Infection: M01.X3, M01.X4, M01.X39, M01.X31, M00.031, M00.032, M00.039 M00.041, M00.042, M00.049 M00.231, M00.232, M00.239, M00.241, M00.242, M00.249, M00.831, M00.832, M00.839, M00.841, M00.842, M00.849, M71.031, M71.032, M86.031, M86.032, M86.039, M86.041, M86.042, M86.049, M86.131, M86.132, M86.139, M86.141, M86.142, M86.149, M86.231, M86.232, M86.239, M86.241, M86.242, M86.249, M86.331, M86.332, M86.339, M86.341, M86.349, M86.431, M86.432, M86.439, M86.441, M86.442, M86.449, M86.531, M86.532, M86.539, M86.541, M86.542, M86.549, M86.631, M86.632, M86.639, M86.641, M86.642, M86.649, M86.8X3, M86.8X7

Trauma: M87.233, M87.236, M87.243, M87.23, M87.237, M87.238, M87.231, M87.231, M87.235, M87.241, M87.242, M87.24, M87.23, M12.531, M12.532, M19.131, M19.132, M19.139, M12.539, S61.401A, S61.401S, S61.402A, S61.402S, S61.409A, S61.409S, S61.411A, S61.411S, S61.412A, S61.412S, S61.419A, S61.419S, S61.421A, S61.421S, S61.422A, S61.422S, S61.431A, S61.431S, S61.431S, S61.432A, S61.432S, S61.439A, S61.439S, S61.441A, S61.441S, S61.442A, S61.442S, S61.449A, S61.449S, S61.451A, S61.451S, S61.452A, S61.452S, S61.459A, S61.459S, S61.5, S62.0, S62.1, S62.2, S62.5, S62.9,

© 2025 Schoenfeld AJ et al. *JAMA Network Open*.

S62.001A, S62.001B, S62.001S, S62.002A, S62.002B, S62.002S, S62.009A, S62.009B, S62.009S, S62.011A, S62.011B, S62.011S, S62.012A, S62.012B, S62.012S, S62.013A, S62.013B, S62.013S, S62.014A, S62.014B, S62.014S, S62.015A, S62.015B, S62.015S, S62.016A, S62.016B, S62.016S, S62.021A, S62.021B, S62.021S, S62.022A, S62.022B, S62.026S, S62.023A, S62.023B, S62.023B, S62.023S, S62.024A, S62.024B, S62.024S, S62.025A, S62.025B, S62.025S, S62.026A, S62.026B, S62.026S, S62.031A, S62.031B, S62.031S, S62.032A, S62.032A, S62.032B, S62.032S, S62.033A, S62.033B, S63.033S, S62.034A, S62.034B, S62.034S, S62.035A, S62.035B, S62.035S, S62.036A, S62.036B, S62.036S, S62.101A, S62.101B, S62.101S, S62.102A, S62.102B, S62.102S, S62.109A, S62.109B, S62.109S, S62.111A, S62.111B, S62.111S, S62.113A, S62.113B, S62.113S, S62.114A, S62.114B, S62.114S, S62.115A, S62.115B, S62.115S, S62.116A, S62.121A, S62.121B, S62.121S, S62.122A, S62.122B, S62.122S, S62.124A, S62.124B, S62.124S, S62.125A, S62.125B, S62.125S, S62.126A, S62.126B, S62.126S, S62.131A, S62.131B, S62.131S, S62.132A, S62.132B, S62.132S, S62.133A, S62.133B, S62.133S, S62.134A, S62.134B, S62.134S, S62.135A, S62.135B, S62.135S, S62.136A, S62.136B, S62.136S, S62.141A, S62.141B, S62.141S, S62.142A, S62.142S, S62.143A, S62.143B, S62.143S, S62.144A, S62.144B, S62.144S, S62.145A, S62.145B, S62.145S, S62.146A, S62.146B, S62.146S, S62.151A, S63.151B, S62.151S, S62.152A, S62.152B, S62.152S, S62.153A, S62.153B, S62.153S, S62.154A, S62.154B, S62.154S, S62.155A, S62.155B, S62.155S, S62.156A, S62.156B, S62.156S, S62.161A, S62.161B, S62.161S, S62.162A, S62.162B, S62.162S, S62.163A, S62.163B, S62.163S, S62.164A, S62.164B, S62.164S, S62.165A, S62.165B, S62.165S, S62.166A, S62.166B, S62.166S, S62.171A, S62.171B, S62.171S, S62.172A, S62.172B, S62.172S, S62.173A, S62.173B, S62.173S, S62.174A, S62.174B, S62.174S, S62.175A, S62.175B, S62.175S, S62.176A, S62.176.B, S62.176S, S62.181A, S62.181B, S62.181S, S62.182A, S62.182B. S62.182S, S62.183A, S62.183B, S62.183S, S62.184A, S62.184B, S62.184S, S62.185A, S62.185B, S62.185S, S62.186S, S62.201A, S62.201B, S62.201S, S62.202A, S62.202B, S62.202S, S62.209A, S62.209B, S62.209S, S62.211A, S62.211B, S62.211S, S62.212A, S62.212B, S62.212S, S62.213A, S62.213B, S62.213S, S62.221A, S62.221B, S62.221S, S62.222A, S62.222B, S62.222S, S62.223A, S62.223B, S62.223S, S62.224A, S62.224B, S62.224S, S62.225A, S62.225B, S62.225S, S62.226A, S62.226B,S62.226S, S62.231A, S62.231B, S62.231S,S62.233A, S62.233B, S62.233S, S62.234A, S62.234B, S62.234S, S62.235A, S62.235B, S62.235S, S62.236A, S62.236B, S62.236S, S62.241A, S62.241B, S62.241S, S62.242A, S62.242B, S62.242S, S62.243A, S62.243B, S62.243S, S62.244A, S62.245A, S62.245B, S62.245S, S62.291A, S62.291B, S62.291S, S62.292A, S62.292B, S62.292S, S62.299A,S62.299A, S62.299B, S62.299S, S62.90XA, S62.90XB, S62.90XS, S62.91XA, S62.91XB, S62.91XS, S62.92XA, S63.3 S62.92XB, S62.92XS, S63.501A, S63.502A, S63.509A, S63.522A, S63.521A, S63.529A, S63.599A, S63.90XS, S63.8X9A, S63.90XA, S63.519A, S63.8X1A, S63.8X2A, S63.91XA, S63.92XA, S63.301, S63.3, S63.0, S63.9, S63.5, S68.4, S68.7, S58.1, S67.32XA, S67.31XA, S67.31XS, S67.32XS, S67.40XA, S67.40XS, S67.41XA, S67.41XS, S67.42XA, X67.42XS, S64, S65, S66, S69.80XA, S69.80XS, S69.81XA, S69.81XS, S69.82XA, S69.81XS, S69.82XA, S69.82XS, S69.90XA, S69.90XS, S69.91XA, S69.91XS, S69.92XA, S69.92XS, S68, S65, S66, T79.A11A, T79.A11S, T79.A12A, T79.A12S, T79.A19A, T79.A19S

© 2025 Schoenfeld AJ et al. *JAMA Network Open*.

Tumor: C49.10, C49.11, C49.12, C40.00, C40.01, C40.02, C40.10, C40.11, C40.12, C40.12, D21.10, D21.11, D21.12, M84.539S, M84.539A, M83.534A, M83.534S M83.533A, M83.533S, M84.541A, M84.541S, M84.542S, M84.549A, M84.549S, D36.12, D16.10, D16.11, D16.12, D16.00, D16.01, D16.02

Ankle Arthroscopy

Infection: M01.X71, M01.X72, M01.X79, M86.061, M86.062, M86.069, M86.071, M86.072, M86.079, M86.161, M86.162, M86.169, M86.171, M86.172, M86.179, M86.261, M86.262, M86.269, M86.271, M86.272, M86.279, M86.361, M86.362, M86.369, M86.371, M86.372, M86.379, M86.461, M86.462, M86.469, M86.471, M86.472, M86.479, M86.561, M86.561, M86.562, M86.569, M86.571, M86.572, M86.579, M86.661, M86.662, M86.669, M86.671, M86.679, M86.8X6, M86.8X7, M00.071, M00.0.072, M00.079, M00.171, M00.172, M00.179, M00.271, M00.272, M00.279, M00.871, M00.872, M00.879, M60.070, M60.071, M60.072, L08.9, T84.59XA, M71.071, M71.072, M71.079, M65.071, M65.072, M65.079, M00.871, M00.872, M00.879

Trauma: M87.271, M87.272, M87.273, M87.276, M12.579, M61.071, M61.072, M61.079, M12.571, M12.572, S82.301A, S82.3, S82.301B, S98, S82.301C, S82.301S, S82.302A, S82.302B, S82.302C, S82.302S, S82.309A, S82.309B, S82.309C, S82.309S, S82.311A, S82.311S, S82.312A, S82.312S, S82.319A, S82.319S, S82.391A, S82.391B, S82.391C, S82.391S, S82.392A, S82.392B, S82.392C, S82.392S, S82.399A, S82.399B, S82.399C, S82.399S, S82.6, S82.61XA, S82.61XB, S82.61XC, S82.61S, S82.62XA, S82.62XB, S82.62XC, S82.62XS, S82.63XA, S82.63XB, S82.63XC, S82.63XS, S82.64XA, S82.64XB, S82.64XC, S82.64XS, S82.65XA, S82.65.XB, S82.65XC, S82.65XS, S82.66XA, S82.66XB, S82.66XC, S82.66XS, S82.8, S82.821A, S82.821S, S82.822A, S82.822S, S82.829A, S82.829S, S82.831A, S82.831B, S82.831C, S82.831S, S82.841A, S82.841B, S82.841C, S84.841S, S82.842A, S82.842B, S82.842C, S82.842S, S82.843A, S82.843B, S82.843C, S82.843S, S82.844A, S82.844B, S82.844C, S82.844S, S82.845A, S82.845B, S82.845C, S82.845S, S82.846A, S82.846B, S82.846C, S82.846S, S82.851A, S82.851B, S82.851C, S82.851S, S82.852A, S82.852B, S82.852C, S82.852S, S82.853A, S82.853B, S82.853C, S82.853S, S82.854A, S82.854B, S82.854C, S82.854S, S82.855A, S82.855B, S82.855C, S82.855S, S82.856A, S82.856B, S82.856C, S82.856S, S82.861A, S82.861B, S82.861C, S82.861S, S82.862A, S82.862B, S82.862C, S82.862S, S82.863A, S82.863B, S82.863C, S82.863S, S82.864A, S82.864B, S82.864C, S82.864S, S82.865A, S82.865B, S82.865C, S82.865S, S82.866A, S82.866B, S82.866C, S82.866S, S82.871A, S82.871B, S82.871C, S82.871S, S82.872A, S82.872B, S82.872C, S82.872S, S82.873A, S82.873B, S82.873C, S82.873S, S82.874A, S82.874B, S82.874C, S82.874S, S82.875A, S82.875B, S82.875C, S82.875S, S82.876A, S82.876B, S82.876C, S82.876S, S82.51XA, S82.51XB, S82.51XC, S82.51XS, S82.52XA, S82.52XB, S82.52XC, S82.52XS, S82.53XA, S82.53XB, S82.53XC, S82.53XS, S82.54XA, S82.54XB, S82.54XC, S82.54XS, S82.55XA, S82.55XB, S82.55XC, S82.55XS, S82.56XA, S82.56XB, S82.56XC, S82.56XS, S92, S92.101A, S92.101B, S92.101S, S92.102A, S92.102B, S92.102S, S92.109A, S92.109B, S92.109S, S92.111A, S92.111B, S92.111S, S92.113A, S92.113B, S92.113S, S92.114A, S92.114B, S92.114S, S92.115A, S92.115B,

© 2025 Schoenfeld AJ et al. *JAMA Network Open*.

S92.115S, S92.116A,S92.116B, S92.116S, S92.121A, S92.121B, S92.121S, S92.122A, S92.122B, S92.122S, S92.123A, S92.123B, S92.123S, S92.124A, S92.124B, S92.124S, S92.125A, S92.125B, S92.125S, S92.126A, S92.126B, S92.126S, S92.131A, S92.121B, S92.131S, S92.132A, S92.132B, S92.132S, S92.133A, S92.133B, S92.133S, S92.134A, S92.135A, S92.135B, S92.135S, S92.136A, S92.136B, S92.136S, S92.141A, S92.141B, S92.101S, S92.142A, S92.142B, S92.142S, S92.143A, S92.143B, S92.143S, S92.144A, S92.144B, S92.144S, S92.145A, S92.145B, S92.145S, S92.146A, S92.146B, S92.146S, S92.151A, S92.151B, S92.151S, S92.152A, S92.152B, S92.152S, S92.153A, S92.153B, S92.153S, S92.154A, S92.154B, S92.154S, S92.155A S92.155B, S92.155S, S92.156A, S92.156B, S92.156S, S92.191A, S92.191B, S92.191S, S92.192A, S92.192B, S92.192S, S92.199A, S92.199B, S92.199S, S93.4, S93.01XA, S93.01XS, S93.03XA, S93.03XS, S93.04XA, S93.04S, S93.05XA, S93.05XS, S93.06XA, S93.06XS, S93.401A, S93.401S, S93.402A, S93.402S, S93.409A, S93.409S, S93.411A, S93.411S, S93.412A, S93.412S, S93.419A, S93.419S, S93.421A, S93.421S, S93.422A, S93.422S, S93.429A, S93.429S, S93.431A, S93.431S, S93.432A, S93.432S, S93.439A, S93.439S, S93.491A, S93.491S, S93.492A, S93.492S, S93.499A, S93.499S, S93, S94, S97.00XA, S97.00XS, S97.01XA, S97.01XS, S97.02XA, S97.02XS, S99, S91.001A, S91.001S, S91.002A, S91.002S, S91.011A, S91.011S, S91.012A, S91.012S, S91.019A, S91.019S, S91.021A, S91.021S, S91.022A, S91.022S, S91.029A, S91.029S, S91.031A, S91.031S, S91.032A, S91.032S, S91.039A, S91.039S, S91.041A, S91.041S, S91.042A, S91.042S, S91.051A, S91.051S, S91.052A, S91.052S, S91.059A, S91.059S, S94.00XA, S94.00XS, S94.01XA, S94.01XS, S94.02XA, S94.02XS, S94.10XA, S94.10XS, S94.11XA, S94.11XS, S94.12XA, S94.12XS, S94.20XA, S94.20XS, S94.21XA, S94.21XS, S94.22XA, S94.22XS, S94.30XA, S94.30XS, S94.31XA, S94.31XS, S94.32XA, S94.32XS, S94.8X1A, S94.8X1S, S94.8X2A, S94.8X2S, S94.90XA, S94.90XA, S94.90XS, S94.91XA, S94.91XS, S94.92XA, S94.92XS, S95, S96

Tumor: M90.671, M90.672, M90.679, M84.571A, M84.571S, M84.572A, M84.572S, M84.573A, M84.573S, M84.574A, M84.574S, M84.575A, M84.575A, M84.561A, M84.561S, M84.562A, M84.562S, M84.563A, M84.563S, M84.564A, M84.564S, M84.569A, M84.569S, C40.80, C40.81, C40.82, C40.90, C40.91, C40.92, C40.20, C40.21, C40.22, C40.30, C40.31, C40.32, D21.21, D21.22, D36.13, D16.20, D16.21, D16.22, D16.30, D16.31, D16.32

© 2025 Schoenfeld AJ et al. *JAMA Network Open*.
