# Supplementary material for: Surgical Low-Value Care Between Fee-For-Service and Salaried Health Care Systems
Source: JAMA Netw Open. 2025 Dec 2;8(12):e2546213. doi: 10.1001/jamanetworkopen.2025.46213 (PMC12673412; doi:10.1001/jamanetworkopen.2025.46213)
Supplement: Supplement 2. — Data Sharing Statement [file jamanetwopen-e2546213-s002.pdf]

Data Sharing Statement

Schoenfeld. Surgical Low-Value Care Between Fee-For-Service and Salaried Health Care Systems. *JAMA Netw Open*. Published December 02, 2025. doi:10.1001/jamanetworkopen.2025.46213

Data
Data available: No

Additional Information
Explanation for why data not available: Data is proprietary to the DoD
